# Five-year remission without disease progression in a patient with relapsed/refractory multiple myeloma with extramedullary disease treated with LCAR-B38M chimeric antigen receptor T cells in the LEGEND-2 study: a case report

**DOI:** 10.1186/s13256-022-03636-9

**Published:** 2022-12-11

**Authors:** Bai-Yan Wang, Wan-Hong Zhao, Yin-Xia Chen, Xing-Mei Cao, Yun Yang, Yi-Lin Zhang, Fang-Xia Wang, Peng-Yu Zhang, Bo Lei, Liu-Fang Gu, Jian-Li Wang, Ju Bai, Yan Xu, Xu-Geng Wang, Rui-Li Zhang, Li-Li Wei, Qiu-Chuan Zhuang, Frank Fan, Wang-Gang Zhang, Ai-Li He, Jie Liu

**Affiliations:** 1 Department of Hematology, The Second Affiliated Hospital of Xi’an Jiaotong University, 157 Xiwu Road, Xincheng District, Xi’an, 710004 China; 2 The Second Affiliated Hospital of Xi’an Jiaotong University, Xi’an, China; 3 Nanjing Legend Biotech, Nanjing, China; 4 Department of Hematology and National-Local Joint Engineering Research Center of Biodiagnostics & Biotherapy, The Second Affiliated Hospital of Xi’an Jiaotong University, Xi’an, China

**Keywords:** Multiple myeloma, CAR-T, BCMA, Case study, Extramedullary disease

## Abstract

**Background:**

Multiple myeloma remains incurable despite treatment advancements over the last 20 years. LCAR-B38M Cells in Treating Relapsed/Refractory Multiple Myeloma was a phase 1, first-in-human, investigator-initiated study in relapsed/refractory multiple myeloma conducted at four sites in China. The study used LCAR-B38M chimeric antigen receptor-T cells expressing two B-cell maturation antigen-targeting single-domain antibodies designed to confer avidity, and a CD3ζ signaling domain with a 4-1BB costimulatory domain to optimize T-cell activation and proliferation. This chimeric antigen receptor construct is identical to ciltacabtagene autoleucel. In the LEGEND-2 study (*n* = 57, Xi’an site), overall response rate was 88%; median (95% CI) progression-free survival and overall survival were 19.9 (9.6–31.0) and 36.1 (26.4–not evaluable) months, respectively; and median follow-up was 25 months. This case study reports on a patient with relapsed/refractory multiple myeloma (λ light chain type) who was treated with LCAR-B38M chimeric antigen receptor T cells in the LEGEND-2 study (Xi’an site); he had received five prior lines of treatment and had extensive extramedullary lesions.

**Case presentation:**

The patient, a 56-year-old Asian male, received cyclophosphamide (500 mg daily × 3 days) as lymphodepletion therapy and a total dose of 0.5 × 10^6^ chimeric antigen receptor + T cells/kg split into three infusions (days 1, 24, and 84 from June to August 2016). He experienced grade 2 cytokine release syndrome after the first infusion; all symptoms resolved with treatment. No cytokine release syndrome occurred following the second and third infusions. His λ light chain levels decreased and normalized 20 days after the first infusion, and extramedullary lesions were healed as of January 2018. He has sustained remission for 5 years and received no other multiple myeloma treatments after LCAR-B38M chimeric antigen receptor T cell infusion. As of 30 October 2020, the patient is still progression-free and has maintained minimal residual disease-negative (10^–4^) complete response status for 52 months.

**Conclusions:**

This case provides support that treatment with LCAR-B38M chimeric antigen receptor T cells can result in long-term disease remission of 5 or more years without disease progression in a heavily pretreated patient with extensive extramedullary disease and no other treatment options.

## Background

Multiple myeloma (MM) is a genetically complex and heterogeneous malignancy affecting antibody-producing plasma cells [1, 2] and is associated with immune surveillance evasion, suppression of T-cell functions, and other immunosuppressive effects [3]. Patients with MM experience substantial morbidity and mortality [4]; while the introduction of new treatment options in the last 20 years has extended the median survival of patients diagnosed with MM [3], the survival rate at 5 years is only about 50% [5]. As the disease progresses, each subsequent line of treatment a patient with MM receives is associated with shorter progression-free survival, decreased rate, depth, and durability of response, as well as decreased quality of life [6–8]. Hence, MM remains incurable because of the heterogeneity of this hematological malignancy [1, 2], and most patients eventually relapse. Even in the era of novel agents, MM complicated by increased International Staging System (ISS) stage, high-risk cytogenetics, and extramedullary disease remains a condition associated with poor prognosis and drug resistance [9, 10]. Multiple studies have shown that patients with MM and extramedullary disease have shorter progression-free survival and overall survival [10–13].

Chimeric antigen receptor (CAR)-T cell therapy is a novel MM treatment using autologous T-cells that are modified to target and kill malignant plasma cells [14]. Several CAR-T cell therapies targeting B-cell maturation antigen (BCMA), which has a highly plasma cell-specific expression pattern, have demonstrated promising efficacy in patients with relapsed/refractory (R/R) MM, including those with extramedullary disease [15–18]. LCAR-B38M is a CAR-T cell therapy containing cells expressing two B-cell maturation antigen (BCMA)-targeting single-domain antibodies designed to confer avidity, and a CD3ζ signaling domain with a 4-1BB costimulatory domain to optimize T-cell activation and proliferation. This CAR construct is identical to ciltacabtagene autoleucel [19]. LCAR-B38M Cells in Treating Relapsed/Refractory Multiple Myeloma (LEGEND-2), a first-in-human, phase 1, investigator-initiated, open-label study, found that LCAR-B38M had a manageable safety profile and led to deep and durable responses in patients with R/R MM [20–22]. This is the first case report from the LEGEND-2 study describing a heavily pretreated patient with extensive extramedullary disease who had long-term remission after receiving LCAR-B38M CAR-T cells.

## Case presentation

A 56-year-old Asian male is the subject of this case. He was a farmer who lived locally and had no history of travel abroad. His wife, son, and daughter were in good health. The patient was previously healthy with no history of hepatitis, tuberculosis, heart disease, diabetes, trauma, or allergies, and he received no medication prior to diagnosis. He smoked approximately 20 cigarettes per day for > 30 years and had no history of alcohol addiction.

The patient initially presented to the urology department in early March 2014 with left testis enlargement. Uneven bilateral testicular echo and enlarged left testis accompanied by epididymal cyst were indicated by B-mode ultrasound. A 3 × 4 cm painless mass below the xiphoid was identified upon physical examination. Bilateral orchiectomy was performed, and the pathology indicated extramedullary plasmacytoma. Bone marrow smear showed that immature plasma cells accounted for 2% of bone marrow cellularity. In April 2014, the patient was diagnosed with active MM (λ light chain type), as defined by International Myeloma Working Group criteria. The patient met Durie/Salmon stage IIIA [23] and ISS stage I [24] criteria, demonstrating clear evidence of disease. Fluorescence *in situ* hybridization for detection of high-risk cytogenetic abnormalities was not performed. The patient had no prior history of any other hematologic malignancy.

The mass below the xiphoid resolved following one cycle of vinorelbine, pirarubicin, and dexamethasone (VAD). Subsequently, the patient received five cycles of VAD as consolidation chemotherapy; one cycle of cisplatin, etoposide, ifosfamide, and dexamethasone (DCEP); one cycle of lenalidomide, cyclophosphamide, and dexamethasone (RCD); and then lenalidomide 10 mg once daily as maintenance therapy. Around April 2016, multiple painless nodules appeared on the skin of the patient’s head, face, trunk, and extremities.

The bone marrow aspirate smear showed that plasma cells accounted for only 1% of the bone marrow cellularity; however, relapse criteria were met with the nodules and with urine λ light chain concentration of 234 mg/L, well above the normal range (NR) of < 3.78 mg/L. No other abnormalities were identified, and the patient was diagnosed with disease progression. The VAD regimen was administered repeatedly, and the nodules on the whole body increased compared with the previous period. Fludarabine and cyclophosphamide were given; however, the nodules were not significantly reduced.

**Fig. 1:**
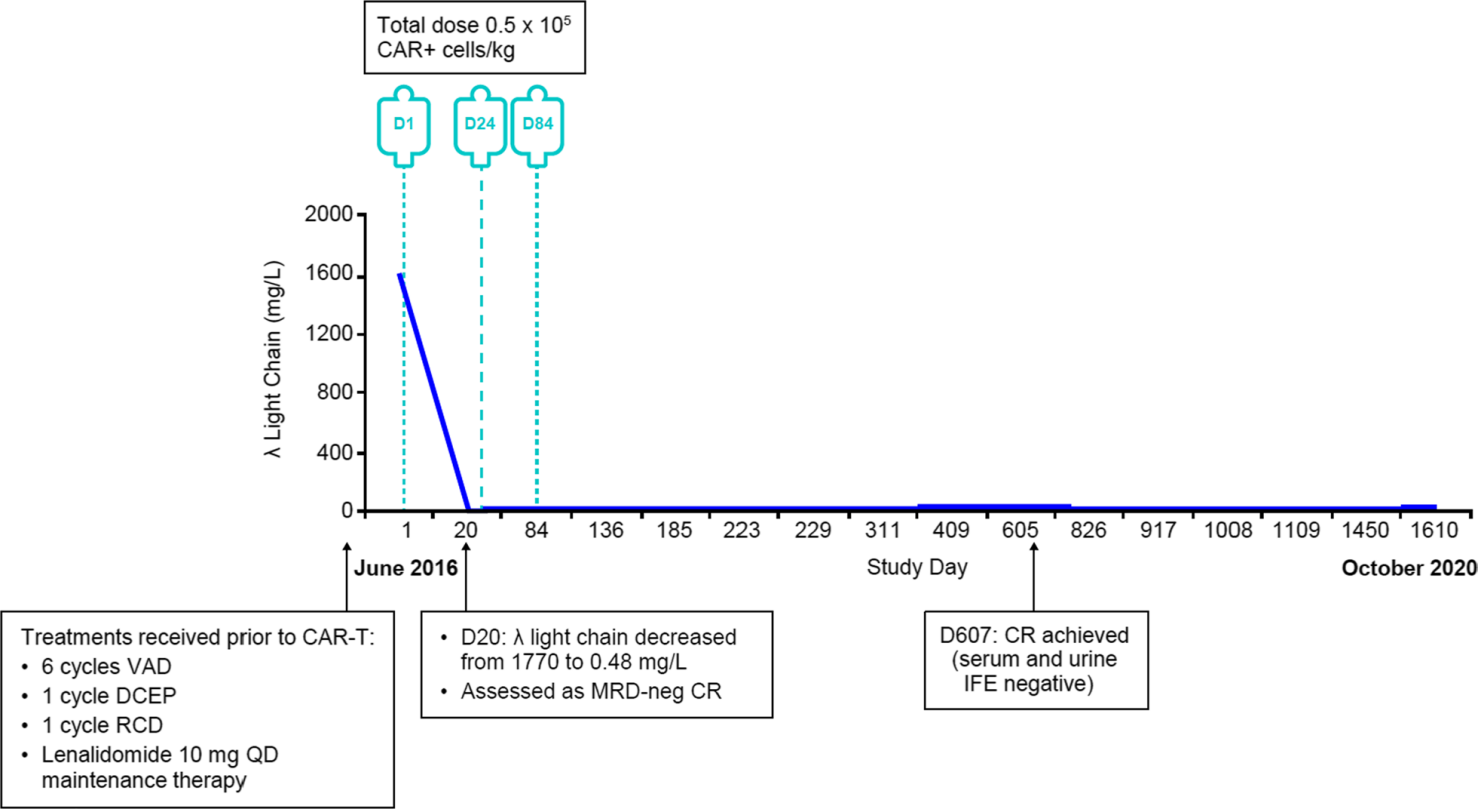
Treatment with LCAR-B38M CAR-T cell therapy resulted in rapid λ light chain decline. Patient’s λ light chain levels (mg/L) prior to receiving LCAR-B38M CAR-T cells and throughout long-term follow-up to October 2020. CAR, chimeric antigen receptor; CR, complete response; DCEP, cisplatin, etoposide, ifosfamide, and dexamethasone; IFE, immunofixation; MRD, minimal residual disease; QD, once daily; RCD, lenalidomide, cyclophosphamide, and dexamethasone; VAD, vinorelbine, pirarubicin, and dexamethasone

The patient was determined to be eligible to receive CAR-T cell treatment and following informed consent from both the patient and his family, he was enrolled in the LEGEND-2 study (Xi’an site; NCT03090659) in May 2016. Baseline bone marrow plasma cells were 8.0% (24 May 2016), and serum-free λ light chain was 1770 mg/L (29 April 2016; NR: 8–27 mg/L; Fig. 1). He was given cyclophosphamide 500 mg daily as lymphodepletion therapy for 3 days before receiving LCAR-B38M CAR-T cell therapy.

On 4 June 2016, he received the first LCAR-B38M CAR-T cell infusion (20 × 10^6^ total viable T cells; 7.76 × 10^6^ total viable CAR-T cells; 0.13 × 10^6^/kg CAR-T cells), lasting for approximately 5 minutes, with no complaints of discomfort from the patient. Prior to infusion, his vital signs were normal [temperature: 36.2 °C; pulse: 78 beats per minute; respiration: 19 breaths per minute; blood pressure: 110/65 mmHg]. He had no fever, cough, nausea, or vomiting; was in a good mental state; and had poor appetite. Urine and feces were also normal. Physical examination showed multiple painless nodules on the skin throughout the body, and enlarged lymph nodes were palpable in the left neck and left groin. The nodular surface of the right side of the patient’s face was partially ulcerated. Following infusion, the residual cell suspension was applied to these ulcerated nodules.

Ten days after the first infusion, on 14 June 2016, the patient developed fever with dyspnea and eyelid edema. He was treated with antipyretics, and the fever was unresponsive. Laboratory results on the same day showed normal liver and renal functions, but activated partial thromboplastin time was elevated (Table 1). The next day, he was admitted to the hospital emergency department. The electrocardiogram (ECG) showed sinus arrhythmia, atrial premature beats, and elevated heart rate (180 beats per minute). His cytokine profile revealed grade 2 cytokine release syndrome (CRS). He was treated with ibuprofen, dexamethasone, and tocilizumab for CRS and pyrexia; piperacillin/tazobactam for infection prophylaxis; bisoprolol for rapid atrial fibrillation; supplemental oxygen therapy; granulocyte colony-stimulating factor for neutropenia; and diuretics for reducing heart burden and maintaining a stable internal environment; in addition, he was placed under ECG monitoring. His body temperature returned to normal on the same day of admission. On 16 June 2016, all symptoms had resolved. His cytokine profile was monitored for an additional 10 days; his cytokine levels decreased slowly over time. The patient underwent a 24-hour Holter ECG on 21 June 2016, which indicated atrial fibrillation accompanied by a paroxysmal rapid ventricular rate, with a maximum heart rate of 131 beats per minute. Propafenone was given, and the arrhythmia and premature beats resolved.

**Table 1:** Patient monitoring characteristics during cytokine release syndrome adverse event

Characteristic/date	14 June 2016	15 June 2016	16 June 2016	20 June 2016	27 June 2016
Peak body temperature, °C	39	38.9	36.7	–	–
Activated partial thromboplastin time, seconds (NR)	43 (23–35)	–	–	Within the normal range	–
Heart rate, beats per minute	–	180	88	–	–
*Cytokine profile*					
hIL-6, pg/mL (NR < 3.4)	–	314.00	–	37.40	27.40
hIL-8, pg/mL (NR < 14)	–	92.70	–	133.00	26.20
hIL-10, pg/mL (NR < 9.1)	–	392.00	–	50.30	Normal
TNF-α, pg/mL (NR < 8.1)	–	52.10	–	42.50	22.00

h, human; IL, interleukin; NR, normalized ratio; TNF, tumor necrosis factor

The patient received the second LCAR-B38M CAR-T cell infusion on 27 June 2016 (20 × 10^6^ total viable T cells; 7.76 × 10^6^ total viable CAR-T cells; 0.13 × 10^6^/kg CAR-T cells) and the third LCAR-B38M CAR-T cell infusion on 26 August 2016 (40 × 10^6^ total viable T cells; 15.5 × 10^6^ total viable CAR-T cells; 0.25 × 10^6^/kg CAR-T cells); both the second and third infusions were received without prior lymphodepletion. No adverse events were reported during or after these two infusions. Overall, the patient received a total dose of 0.5 × 10^6^ CAR + T cells/kg split into three infusions on days 1, 24, and 84.

On 23 June 2016, 20 days after the first LCAR-B38M CAR-T cell infusion, serum free λ light chain was below the lower limit of normal (0.48 mg/L), and the patient’s date of first response was recorded as 24 June 2016; his free light chain levels have since remained within normal limits (Fig. 1). In April and July 2017, bone marrow 8-color flow cytometry (13 probes; 500,000 nucleated cells counted) was negative for abnormal plasma cells. In July 2017, urine immunofixation (IFE) was negative, while serum IFE was indeterminate.

**Fig. 2:**
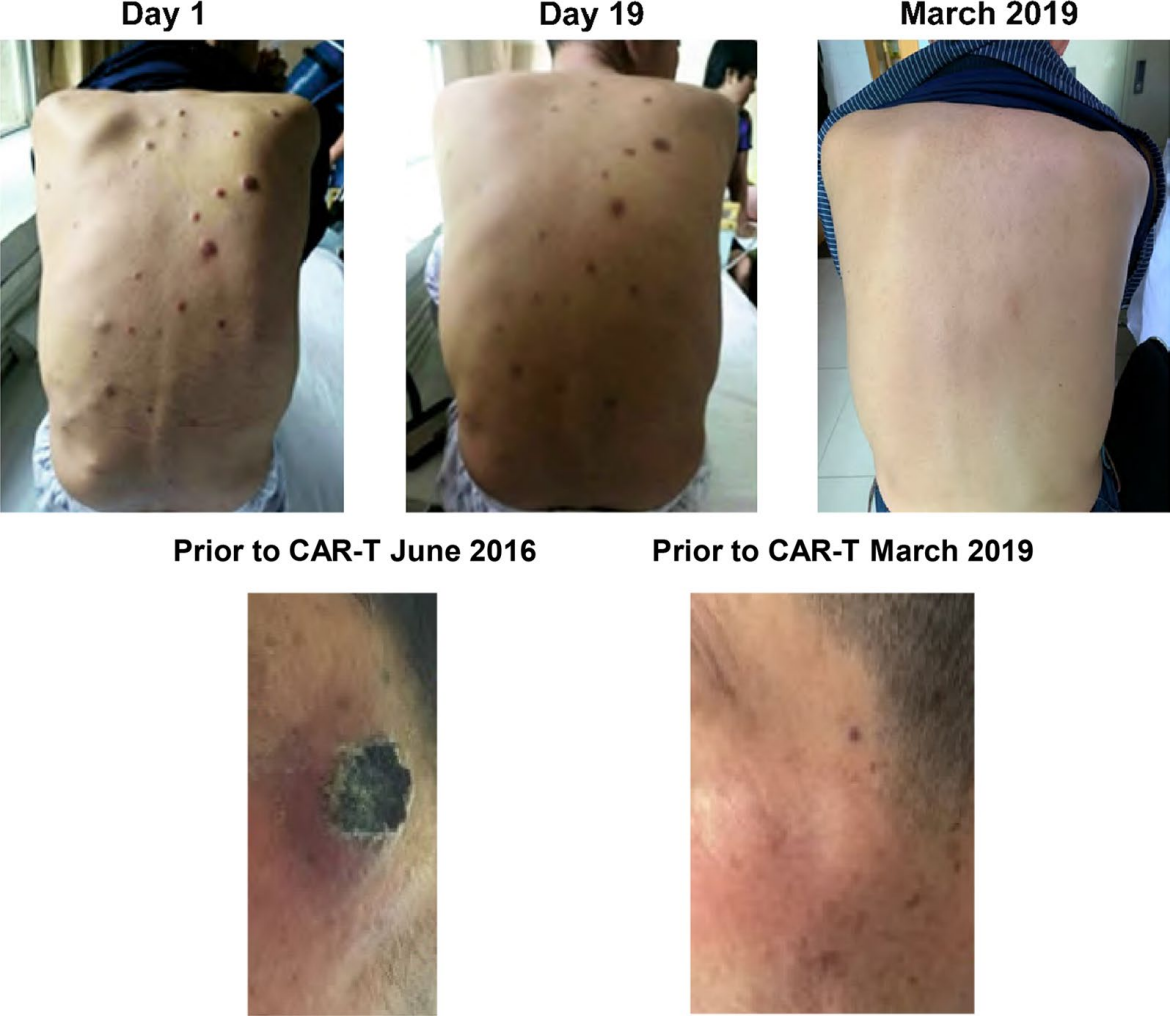
Resolution of extramedullary disease-associated lesions after treatment with LCAR-B38M CAR-T cell therapy. Baseline photo of patient’s extramedullary lesions on day 1 of the LEGEND-2 study (left), on day 19 (center), and after long-term follow-up (33 months) in March 2019 (right) showing clearance of extramedullary lesions. CAR, chimeric antigen receptor

The patient had extensive extramedullary lesions when he enrolled for treatment, which had resolved with or without residue as of 31 January 2018 (20 months post-CAR-T infusion). Since then, his extramedullary lesion-free status has lasted for 33 months (Fig. 2). The patient was assessed as having a complete response on day 607 post CAR-T infusion, since both urine and serum IFE were negative.

**Fig. 3:**
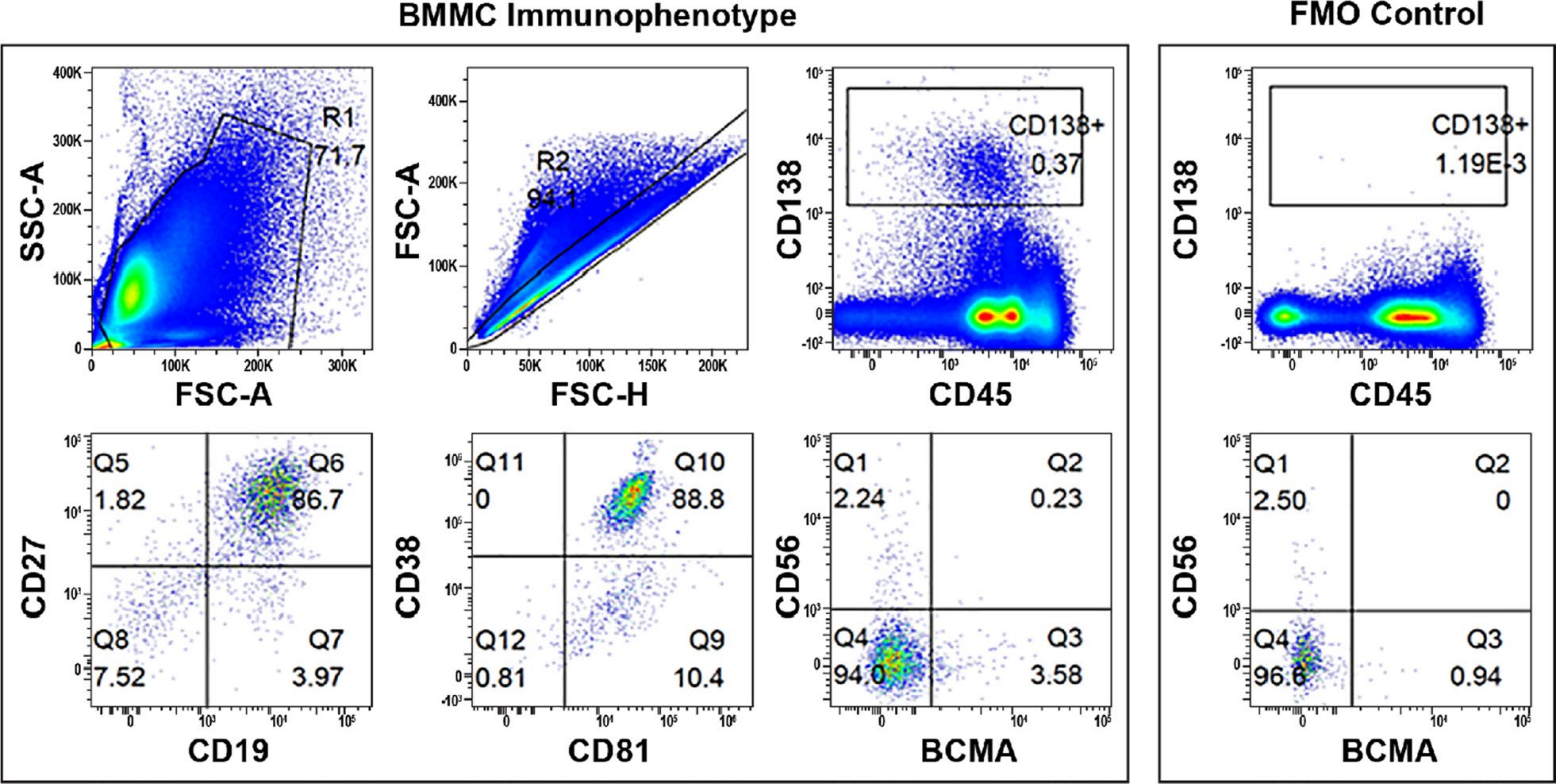
Bone marrow plasma cell immunophenotype by flow cytometry analysis. BCMA, B-cell maturation antigen; BMMC, bone marrow mast cells; FMO, fluorescence minus one

**Fig. 4:**
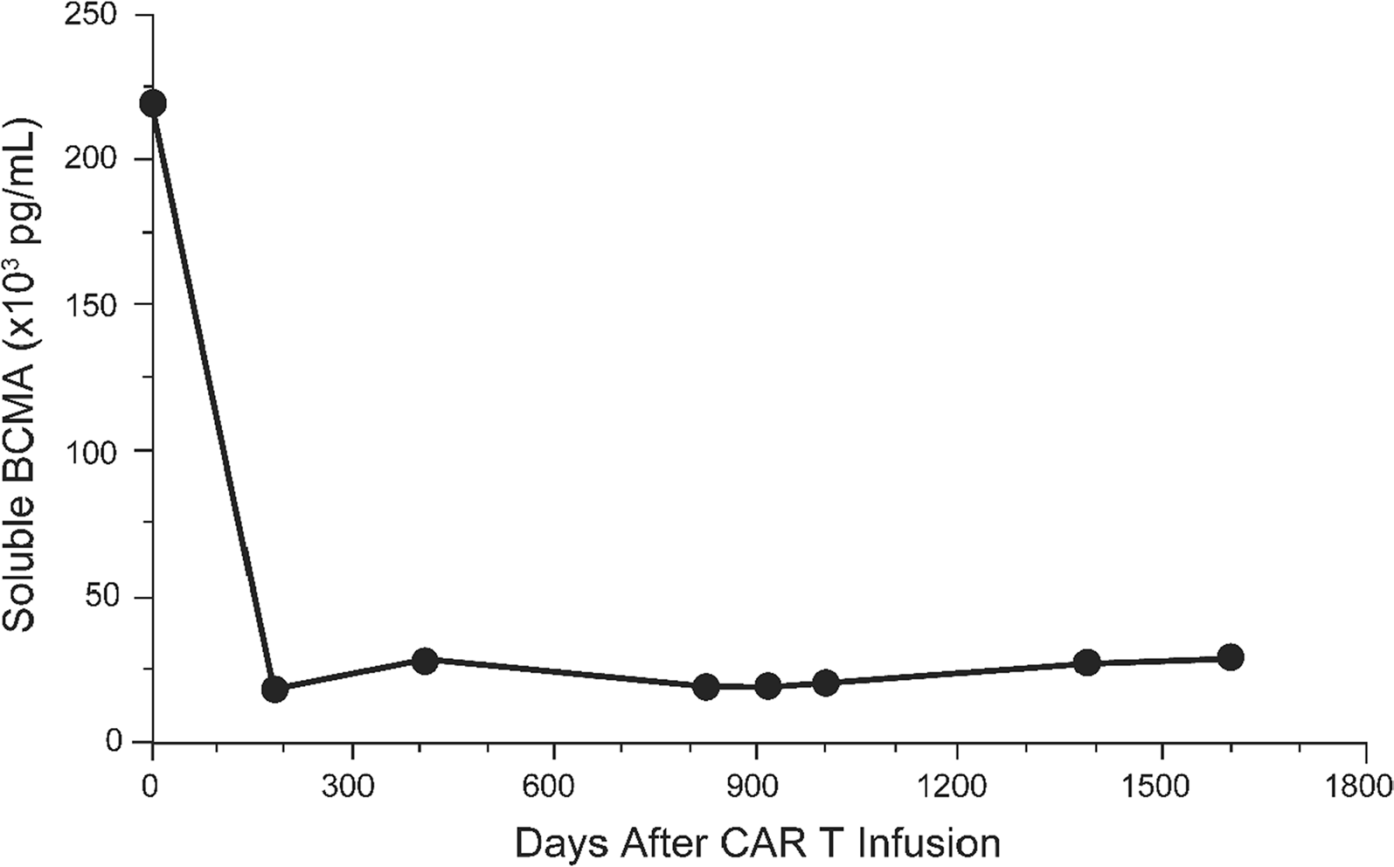
Post-infusion serum soluble BCMA level over time. CAR, chimeric antigen receptor; BCMA, B-cell maturation antigen

After infusion, the CAR transgene was detected at time points over 27 months, and the latest detection was at 825 days. Since then, there has been no detectable CAR transgene or CAR+ T cells in either peripheral blood and bone marrow as of 30 October 2020, which was the latest follow-up visit (4.5 years later). The most recent bone marrow biopsy flow cytometry analysis showed plasma cells with normal immuno-phenotypes CD45+, CD138+, CD19+, CD27+, CD38+, CD81+, CD56−, and BCMA (Fig. 3). After CAR-T cell infusion, the blood soluble BCMA level quickly decreased by more than tenfold within 6 months and has stayed within the normal range for this patient (Fig. 4). No anti-drug antibodies were detected at any time point.

Two 8-color flow cytometry panels were used for assessment of minimal residual disease (MRD), with a sensitivity of 1 × 10^–4^ owing to technical limitations at the time of the study; a total of ten tests were performed 10–52 months post CAR-T infusion, all of which were negative [20]. As of the last follow-up on 30 October 2020, the patient remained progression free and had maintained MRD-negative complete response status. He has had sustained remission for 5 years with no other subsequent treatments for MM after LCAR-B38M CAR-T cell infusion.

## Discussion and conclusions

This 56-year-old Asian male had no history of disease prior to his diagnosis of MM in April 2014. He was at high risk, with light chain MM type and extensive extramedullary lesions at onset, and a high tumor burden at relapse. He had received five prior lines of treatment. The patient was enrolled in the LEGEND-2 clinical trial and received LCAR-B38M CAR-T cells at a total dose of 0.5 × 10^6^ CAR+ T cells/kg split into three infusions between June and August 2016. He experienced grade 2 CRS after the first infusion, which resolved after treatment. The patient first achieved a response 20 days following the first infusion, had complete resolution of extramedullary lesions as of January 2018, and has sustained remission for more than 5 years. This case represents the longest known follow-up of a patient receiving CAR-T cells for the treatment of MM and demonstrates the possibility of long-term remission despite extensive extramedullary disease.

Patients with extramedullary MM are more likely to have a particularly poor outcome [10–13]. Data from the LEGEND-2 study show that LCAR-B38M CAR-T cell therapy is a highly effective treatment in patients with advanced disease, demonstrating an overall response rate of 88% and median progression-free survival of 20 months [25, 26]. This case report of a single patient from the LEGEND-2 study highlights the possibility that LCAR-B38M CAR-T cell therapy may result in a long-term, durable response in a subset of patients with extramedullary disease, with a safety profile that is generally manageable. This patient has a follow-up time of more than 5 years, the longest follow-up of all patients in the first-in-human LEGEND-2 trial [20], and longer than other MM CAR-T cell therapy studies [17, 27, 28]. He has been disease free for several years, despite having multiple extramedullary lesions at baseline. The lesions resolved 20 months post-CAR-T cell treatment, and the resolution has persisted. While not all patients who have received LCAR-B38M CAR-T cell therapy responded as robustly or as long as this patient, the case suggests that a subset of patients may respond very well to CAR-T therapy and maintain the response long term, including those with extramedullary disease. A potential explanation for the deep and durable responses observed in the LEGEND-2 trial, including for this patient, may involve the unique structure of LCAR-B38M. The CAR construct contains two single-domain antibodies targeting separate BCMA epitopes, designed to confer avidity and optimize target engagement.

There are several unique features of this patient. At the time he was treated, the investigators had very limited experience with LCAR-B38M CAR-T cells. Owing to the concern of potential toxicities associated with his high tumor burden, the patient received three infusions over a period of 2 months. Split dosing was not utilized in later trials; it is unclear what effect this may have had on the efficacy or safety of the LCAR-B38M CAR-T therapy.

Most patients in LEGEND-2, including responders and non-responders, had no CAR transgene detectable 6 months after LCAR-B38M CAR-T cell infusion. This patient, however, showed very long CAR transgene persistence, which remained detectable 27 months after LCAR-B38M CAR-T cell infusion. Although it is not clear why CAR transgene persistence was so long in this patient, there is large variability between patients. In CARTITUDE-1, CAR+ T-cell levels and transgene concentrations were highly variable, although they dropped below quantifiable levels by 6 months in a majority of patients [29, 30]. The specific patient factors influencing persistence remain to be elucidated. It is possible that the long-term persistence in this case may have contributed to the sustained response over several years. This case offers support for the efficacy of CAR-T cell therapy in heavily pretreated patients with R/R MM, including those with extramedullary disease.

An expected limitation of this retrospective case study is that several specific laboratory values are missing from the patient’s medical history at diagnosis, including serum levels of λ light chain, β2-microglobulin, and albumin.

Multiple myeloma remains a heterogeneous disorder; many patients become refractory to treatment, and new treatments are needed for patients who progress. Although defining which therapeutic options are superior to others in the R/R MM setting can be difficult (especially in patients with extramedullary disease), in this era of novel agents, there are viable therapeutic options in the majority of cases. These data suggest that LCAR-B38M CAR-T cell therapy is a potentially transformational treatment for patients with R/R MM, a population with limited treatment options and great unmet medical need.

## Data Availability

All data related to the case report are included in this published article.

## Abbreviations

BCMA: B-cell maturation antigen
BMMC: Bone marrow mast cells
CAR: Chimeric antigen receptor
CI: Confidence interval
CR: Complete response
CRS: Cytokine release syndrome
DCEP: Cisplatin, etoposide, ifosfamide, and dexamethasone
ECG: Electrocardiogram
FMO: Fluorescence minus one
h: Human
IFE: Immunofixation
IL: Interleukin
ISS: International Staging System
MM: Multiple myeloma
MRD: Minimal residual disease
NR: Normal range
QD: Once daily
RCD: Lenalidomide, cyclophosphamide, and dexamethasone
R/R: Relapsed/refractory
TNF: Tumor necrosis factor
VAD: Vinorelbine, pirarubicin, and dexamethasone
